# Field assessment of potential exposure of dogs to leptospirosis by measuring antibody titers in dogs: a multisite study in five geographic regions of the United States

**DOI:** 10.3389/fvets.2024.1435630

**Published:** 2024-07-22

**Authors:** Doug Carithers, Ed Loebach, Troy Williams, Jerlyn Sponseller, Andrew Schreibman, Diane Platts

**Affiliations:** ^1^Boehringer Ingelheim, Duluth, GA, United States; ^2^Boehringer Ingelheim, Racine, WI, United States; ^3^Boehringer Ingelheim, Huntersville, NC, United States; ^4^Boehringer Ingelheim, Grafton, MA, United States; ^5^Boehringer Ingelheim, Los Angeles, CA, United States; ^6^Boehringer Ingelheim, League City, TX, United States

**Keywords:** leptospirosis, serovars, MAT titers, unvaccinated, exposure

## Abstract

Leptospirosis vaccine for dogs in the United States is considered a lifestyle or non-core vaccine, making individual veterinary practitioners responsible for determining if vaccination is necessary for their patients. Veterinary professionals often base their vaccination decisions on local rates of clinical cases. However, even subclinical leptospirosis infections have zoonotic potential. The microscopic agglutination test (MAT) is effective for screening unvaccinated animals, but previous vaccination can lead to inconsistent results and variable MAT titers over time. This prospective research survey evaluated if local experience was sufficient to justify selective vaccination for leptospirosis. MAT analyses were performed on sera collected from well-cared-for, unvaccinated dogs residing in five different geographies across the United States: South-Central (East Texas), New England, the Mid-Atlantic (North Carolina and Virginia), Midwest (Wisconsin/northern Illinois), and Southwest (southern California). Thirty-eight clinics participated, submitting a total of 1345 qualified samples from unvaccinated dogs over 1 year of age. 11.6% of these unvaccinated dogs had MAT titers for one or more serogroups of *Leptospira*. While seropositivity does not necessarily indicate that disease will result or that a specific serovar is involved, these MAT-positive cases do indicate that the potential for exposure exists and clinical signs or a carrier-state could result from infection. These survey results would indicate that a more aggressive vaccination protocol for leptospirosis should be considered.

## Introduction

Leptospirosis is a worldwide bacterial spirochetal disease that affects humans (Weil’s Disease, Rat Catcher’s Fever, etc.), animals and is regarded as re-emerging in dogs in North America (1). Infection is spread to dogs either directly or indirectly via contact with the urine of wildlife, farm animals, or other dogs. Leptospirosis can be subclinical or cause a wide range of clinical signs including fever, myalgia, shivering, weakness, lack of appetite, increased thirst and urination, anuria/oliguria, dehydration, vomiting, cough, difficulty breathing, arrhythmias, and/or lymphadenopathy. In some cases, leptospirosis can be associated with multi-organ failure and death (2).

The microscopic agglutination test (MAT) is considered an economically viable reference test for serologic screening, diagnosis of leptospirosis. Sera are screened at 1:100 dilution and, typically, those showing agglutination are serially diluted to determine a titer endpoint. The highest MAT titers were historically considered indicative of the infective serovar, but this can be erroneous because of cross-reactions between serogroups (3). To further complicate the issue, MAT results can be negative in acute infections and do not differentiate vaccinated from infected dogs, making interpretation of results challenging (2).

Previous studies have utilized laboratory animals that are specific pathogen free (4–7) to minimize exposure complications observed with MAT titers. MAT titers were examined in a controlled prospective study using client owned dogs following vaccination (on day 0) and booster (at week 3, ±3 days) with variable MAT titers collected over the span of a year (8), which would be expected to complicate clinical diagnosis of leptospirosis using MAT titers.

The purpose of the current study was to determine *Leptospira* exposure rates in apparently healthy dogs 1 year of age and older, with no history of *Leptospira* vaccination, nor travel history outside their respective geographical regions: South-Central, New England, the Mid-Atlantic, the Midwest, and Southwest. To alleviate complications commonly associated with MAT testing; this prospective research survey assessed the level of exposure to *Leptospira* in well-cared-for, unvaccinated dogs, considered by practitioners to not be at risk of exposure.

## Materials and methods

Forty veterinary clinics agreed to participate in this survey and enroll qualifying patients from dogs brought into the clinic for a wellness examination, targeting 50 dogs for participation per clinic. Eligible dogs were at least 1 year of age, had no current health concerns, had never been vaccinated against leptospirosis, and had not traveled outside of the area of residence.

Prior to inclusion, each pet owner signed a Pet Owner Consent Form (Appendix 1) which gave permission for the clinic staff to draw blood from the dog and send it to the laboratory for testing and acknowledging that the pet owner would not expect to receive results from the survey.

In addition, each clinic filled out the Patient Information Form, providing information including owner name, dog name, breed, age, weight, sex, and sample date on all the enrolled dogs. A sufficient volume of whole blood to secure a 2–3 mL serum sample was collected from each of the qualifying dogs. The serum vials were frozen and retained as they were received and, once all sampling was completed, all samples were submitted to the veterinary diagnostic laboratory at Texas A&M University for serologic testing using the microscopic agglutination test. A total of eight *Leptospira* serovars belonging to the P1 sub-clade of the pathogenic clade were reacted with sera at a 1:100 dilution; no further titrations were performed, as ascertaining potential exposure was the only intent. The seven serogroups used for testing were Pomona, Icterohaemorrhagiae, Canicola, Grippotyphosa, Bratislava, Sejroe (sejroe, hardjo), and Autumnalis, with the respective MAT diagnostic serovar strains used by the laboratory noted in Table 1.

**Table 1:** Serogroups, Serovars, and Serovar strains used for MAT diagnostics.

Serogroup	Pomona	Icterohaemorrhagiae	Canicola	Grippotyphosa	Autumnalis	Sejroe	Australis
Serovar(s)	Pomona	Icterohaemorrhagiae	Canicola	Grippotyphosa	Autumnalis	Sejroe	Bratislava
						Hardjo	
Serovar strain(s)	Pomona (US)	M-20	Hond Ultrecht IV	Andaman	Akiyami A	M-84 (sejroe)	Jez Bratislava
						Hardjoprajtino	

### Statistical methods

Data were analyzed using Fisher’s Exact Test on (nx2) frequency tables. When the overall sample, or one of the regional subsamples, indicated significant (*p* < 0.05) differences between levels of the classification variable, all pairwise combinations of levels were compared using Fisher’s Exact Test.

## Results

A total of 1,363 samples were collected from 38 clinics. Eighteen of those samples were excluded for one of three reasons: poor specimen quality, lack of adequate owner consent, or dog age < 1 year. Of the 1,345 qualifying samples, 156 (11.6%) dogs were seropositive for one or more *Leptospira* serovars.

The Wisconsin/N. Illinois-area (Midwest US) had 55 (14.9%) of 370 dogs testing positive. The New England geography (Northeast US) had 38 (14.6%) of 261 dogs testing positive. The Mid-Atlantic US had 17 (10.8%) of 158 dogs testing positive. The east Texas geography (South-Central US) had 35 (9.1%) of 384 dogs testing positive. The southern California (Southwest US) had 11 (6.4%) of 172 dogs testing seropositive (Table 2).

**Table 2:** Overall results: MAT results, %, total and stat comparisons (1,345 samples from 38 clinics in five geographic regions).

Frequency (Real#/Percent)	Negative	Positive	Total *N*
Overall	1,189	156	1,345
	88.4%	11.6%	
Mid-Atlantic US	141	17	158
	89.2%	10.8%	
Midwest US	315	55	370
	85.1%	14.9%	
Northeast US	223	38	261
	85.4%	14.6%	
South-Central US	349	35	384
	90.9%	9.1%	
Southwest US	161	11	172
	93.6%	6.4%	
Comparison	*p* value		
Overall	0.0100		
Mid-Atlantic vs. Midwest US	0.2568		
Mid-Atlantic vs. Northeast US	0.2981		
Mid-Atlantic vs. South-Central US	0.6303		
Mid-Atlantic vs. Southwest US	0.1707		
Midwest vs. Northeast US	1.0000		
Midwest vs. South-Central US	0.0180		
Midwest vs. Southwest US	0.0046		
Northeast vs. South-Central US	0.0422		
Northeast vs. Southwest US	0.0084		
South-Central vs. Southwest US	0.3210		

In the overall sample of 1,345 dogs, there were significant differences seen among regions. Pairwise comparisons among the regions showed that the South-Central US had a significantly lower rate of positive samples than the Midwest US (*p* = 0.0180) and the Northeast US (*p* = 0.0422); and the Southwest US had a significantly lower rate of positive samples than the Midwest US (*p* = 0.0046) and the Northeast US (*p* = 0.0084) (Table 2).

There was no significant (*p* > 0.10) difference in the rate of positive samples between males and females, either overall or within any of the regions (Table 3).

**Table 3:** Overall results by sex, and results by region, w/stats (females, intact or not vs. males, intact or not).

Frequency (Real#/Percent)	Negative	Positive	Total *N*
Overall			
Female	631	77	708
	89.1%	10.9%	
Male	558	79	637
	87.6%	12.4%	
Overall total	1,189	156	1,345
Mid-Atlantic US			
Female	74	10	84
	88.1%	11.9%	
Male	67	7	74
	90.5%	9.5%	
Mid-Atlantic total	141	17	158
Midwest US			
Female	169	29	198
	85.4%	14.7%	
Male	146	26	172
	84.9%	15.1%	
Midwest total	315	55	370
Northeast US			
Female	99	15	114
	86.8%	13.2%	
Male	124	23	147
	84.4%	15.7%	
Northeast total	223	38	261
South-Central US			
Female	193	18	211
	91.5%	8.5%	
Male	156	17	173
	90.2%	9.8%	
South-Central total	349	35	384
Southwest US			
Female	96	5	101
	95.1%	5.0%	
Male	65	6	71
	91.6%	8.4%	
Southwest total	161	11	172
Comparison		*p* value	
Overall		0.3948	
Mid-Atlantic US		0.7979	
Midwest US		1.0000	
Northeast US		0.6005	
South-Central US		0.7229	
Southwest US		0.3646	

There was no significant (*p* > 0.10) difference in the rate of positive samples among young (1–5 years), middle (>5 to 10 years), or older (>10 years) age groups, either overall or within any of the regions (Table 4).

**Table 4:** Overall results by age group, and results by region (1–5 years, >5 to 10 years, and >10 years).

Frequency (Real#/Percent)	Negative	Positive	Total *N*
Overall			
1–5 years	555	70	625
	88.8%	11.2%	
>5 to 10 years	469	56	525
	89.3%	10.7%	
>10 years	165	30	195
	84.6%	15.4%	
Overall total	1,189	156	1,345
Mid-Atlantic US			
1–5 years	82	9	91
	90.1%	9.9%	
>5 to 10 years	49	6	55
	89.1%	10.9%	
>10 years	10	2	12
	83.3%	16.7%	
Mid-Atlantic total	141	17	158
Midwest US			
1–5 years	155	22	177
	87.6%	12.4%	
>5 to 10 years	115	22	137
	83.9%	16.1%	
>10 years	45	11	56
	84.6%	15.4%	
Midwest total	315	55	370
Northeast US			
1–5 years	89	17	106
	84.0%	16.0%	
>5 to 10 years	92	14	106
	86.8%	13.2%	
>10 years	42	7	49
	85.7%	14.3%	
Northeast total	223	38	261
South-Central US			
1–5 years	179	18	197
	90.9%	9.1%	
>5 to 10 years	134	10	144
	93.1%	6.9%	
>10 years	36	7	43
	83.7%	16.3%	
South-Central total	349	35	384
Southwest US			
1–5 years	50	4	54
	92.6%	7.4%	
>5 to 10 years	79	4	83
	95.2%	4.8%	
>10 years	32	3	35
	91.4%	8.6%	
Southwest total	161	11	172
Comparison		*p* value	
Overall		0.2032	
Mid-Atlantic US		0.7771	
Midwest US		0.3348	
Northeast US		0.8967	
South-Central US		0.1684	
Southwest US		0.7172	

The average weight of the seropositive dogs was 50.5 pounds (range, 7–148 pounds). There were 51 seropositive dogs under 35 pounds, 45 dogs between 35.1 and 60 pounds, and 60 dogs from 60 to 108.6 pounds (Table 5).

**Table 5:** Overall results by weight group, and results by region (<35 lbs., 35.1–60 lbs., and >60 lbs.).

Frequency (Real #/Percent)	Negative	Positive	Total N
Overall			
<35 lbs	528	51	579
	91.2%	8.8%	
35.1–60 lbs	283	45	328
	86.3%	13.7%	
>60 lbs	378	60	438
	86.3%	13.7%	
Overall total	1,189	156	1,345
Mid-Atlantic US			
<35 lbs	64	4	68
	94.1%	5.9%	
35.1–60 lbs	35	4	39
	89.7%	10.3%	
>60 lbs	42	9	51
	82.4%	17.6%	
Mid-Atlantic total	141	17	158
Midwest US			
<35 lbs	127	18	145
	87.6%	12.4%	
35.1–60 lbs	81	13	94
	86.2%	13.8%	
>60 lbs	107	24	131
	81.7%	18.3%	
Midwest total	315	55	370
Northeast US			
<35 lbs	88	9	97
	90.7%	9.3%	
35.1–60 lbs	50	13	63
	79.4%	30.6%	
>60 lbs	85	16	191
	84.2%	15.8%	
Northeast total	223	38	261
South-Central US			
<35 lbs	163	14	177
	92.1%	7.9%	
35.1–60 lbs	92	10	102
	90.2%	9.8%	
>60 lbs	94	11	105
	89.5%	10.5%	
South-Central total	349	35	384
Southwest US			
<35 lbs	86	6	92
	93.5%	6.5%	
35.1–60 lbs	25	5	30
	83.3%	16.7%	
>60 lbs	50	0	50
	100%	0.0%	
Southwest total	161	11	172
Comparison		*p* value	
Overall		0.0186	
<35 lbs vs. 35.1–60 lbs		0.0245	
<35 lbs vs. >60 lbs		0.0148	
35.1–60 lbs vs. >60 lbs		1.0000	
Mid-Atlantic US		0.1163	
Midwest US		0.3802	
Northeast US		0.1182	
South-Central US		0.7232	
Southwest US		0.0106	
<35 lbs vs. 35.1–60 lbs		0.1361	
<35 lbs vs. >60 lbs		0.0903	
35.1–60 lbs vs. >60 lbs		0.0059	

In the overall sample, there were significant differences seen among weight groups. Pairwise comparisons of weight groups showed that the lightest dogs (<35 lbs.) had a significantly lower rate of positive samples than either the medium weight (35.1–60 lbs.; *p* = 0.0245) or the heaviest (>60 lbs.; *p* = 0.0148) dogs. There were no significant differences among weight groups for the Mid-Atlantic, Midwest, Northeast, and South-Central. For the Southwest, large dogs (>60 lbs.) had a significantly (*p* = 0.0059) lower rate of positive samples than the medium weight group (35.1–60 lbs.) (Table 5).

The demographic summary is in Table 6.

**Table 6:** Demographic summary.

Combined	Pos	Neg	All
Dogs	156	1,189	1,345
Ave Age (years)	6.5	6.1	6.2
Ave Weight (lbs.)	50.5	45.3	45.9
Males	78	557	636
Females	78	632	711

Of the 156 seropositive dogs, 92 were MAT positive for one serogroup, 28 were positive for two serogroups, 15 were positive for three serogroups, 15 were positive for four serogroups and six dogs were MAT positive for five serogroups (283 total serovars identified). MAT positive samples were observed for all seven serogroups. 70 dogs positive for Australis serogroup (represented by serovar bratislava), 58 for Grippotyphosa (serovar Grippotyphosa), 56 for Autumnalis (serovar autumnalis), 43 for Pomona (serovar pomona), 40 for Icterohaemorrhagiae (serovar Icterohaemorrhagiae), 14 for Canicola (serovar Canicola), and two for Sejroe (two serovar sejroe, and none for serovar hardjo). Individual sample information for *Leptospira*-positive samples including locations, sample numbers, dog age, weight, sex, and serovars identified are included in Appendix 2.

## Discussion

These data provide an interesting insight into exposure of dogs to *Leptospira* in five separate geographic areas across the United States. In this survey, the dogs were all over 1 year of age and the gender and age incidence was essentially equal. The average age of the dogs included in the study was 6.18 years (range, 1–18 years). The average age of seropositive dogs was 6.54 years of age (range, 1–15 years). Of the 156 positive dogs, 78 females and 78 male dogs seroreacted to one or more of the serovars tested. Weight ranged from 7 lbs. (3.2 kg) to 148 lbs. (67.3 kg). Overall, seropositive dogs were slightly heavier than the average seronegative dog, with a 5.24 lbs. (2.4 kg) weight difference observed in this study.

These data indicated an overall 11.6% rate of exposure of qualifying dogs (healthy dogs with no history of leptospirosis vaccination, over 1 year of age and no history of traveling out of their home area) enrolled in this study from five different regions spanning the United States. The intriguing factor is that veterinarians and/or pet owners chose to not vaccinate the dogs included in this study against leptospirosis. Possible reasons for not vaccinating could be that they felt it was not necessary to vaccinate for leptospirosis, likely expecting little to no opportunity for the dogs to become infected based on their geography or the dog’s lifestyles, or that vaccination posed an unnecessary risk to the dog.

In the South-Central United States, 9.1% of 384 dogs tested positive, in New England 14.6% of 261 dogs tested positive, in the Mid-Atlantic 10.8% of 158 dogs tested positive, in the Midwest 14.9% of 370 dogs tested positive, and in the Southwest 6.4% of 172 dogs tested positive for exposure. Since they were unvaccinated, many would be at risk of developing clinical signs due to pathology consistent with leptospirosis.

In the 156 antibody seropositive dogs, there were 283 hits in all seven of the selected serogroups screened by the diagnostic laboratory. It is important to note that in the United States, available four-way leptospirosis vaccines include Canicola, Grippotyphosa, Icterohaemorrhagiae, and Pomona. Current vaccines can prevent disease resulting from experimental challenge and can decrease shedding of vaccinal serovars (2). These same four vaccine serogroups accounted for 155 of the antibody positive serogroup hits, with three of the four non-canine vaccine serogroups (Australis, Autumnalis, and Sejroe) accounting for 128 antibody positives hits. None of the dogs tested were positive for hardjo antibodies. *Leptospira* hardjo is well-known as a sexually transmitted infection of cattle, often causing abortions, especially in heifers. The *L.* Hardjo MAT was performed simply because it was part of the standard screening panel provided by the diagnostic laboratory, and the authors chose not to add any complications to the normal laboratory procedures.

One must consider that cross-reactivity between serovars within the same serogroup and to serovars in other serogroups has been reported (9, 10). However, for the purposes of this survey, the primary goal was not to identify specific serovars, but to examine the potential Lepto-exposure rate of dogs previously not considered to be at risk.

Seventy dogs harbored antibodies to Australis serogroup. Bratislava is known to cause disease in swine, but there is only limited evidence that bratislava may be a pathogen of dogs in the United States (11). Fifty-six of the antibody hits were for Autumnalis, and two were for Sejroe, both of which have been identified as causing clinical signs of leptospirosis in dogs in the United States, with Sejroe also causing issues in ruminants, and Autumnalis affecting humans and raccoons in the southern US (12). The observations in this serologic survey illustrate dogs are becoming infected, potentially becoming carriers and/or suffering clinical signs, and being involved in the spread of multiple serogroups of *Leptospira* infection. In this survey, it was demonstrated that dogs that were considered no-or low-risk, were seropositive at a rate approximately 10 times higher than nationwide seropositivity for heartworm disease in the United States (13).

These data support the conclusion that perceptions of *Leptospira* exposure risk are likely underestimating the actual risk for dogs across the United States. Further these data support the most recent ACVIM Consensus Statement, where it was stated that “All dogs are at risk of leptospirosis, regardless of signalment, geographic location, lifestyle and the time of year” (2). Thus, an annual vaccination protocol including leptospirosis should be considered for all dogs.

## Data availability statement

The raw data supporting the conclusions of this article will be made available by the authors, without undue reservation.

## Ethics statement

The requirement of ethical approval was waived by Boehringer Ingelheim United States studies for the studies involving animals because samples collected, with pet owner permission, as a service and part of normal veterinary wellness assessments. The studies were conducted in accordance with the local legislation and institutional requirements. Written informed consent was obtained from the owners for the participation of their animals in this study.

## Author contributions

DC: Conceptualization, Data curation, Formal analysis, Funding acquisition, Investigation, Methodology, Project administration, Supervision, Validation, Visualization, Writing – original draft, Writing – review & editing. EL: Investigation, Writing – review & editing. TW: Investigation, Writing – review & editing. JS: Investigation, Writing – review & editing. AS: Investigation, Writing – review & editing. DP: Conceptualization, Investigation, Methodology, Writing – review & editing.
