# Supplementary material for: Field assessment of potential exposure of dogs to leptospirosis by measuring antibody titers in dogs: a multisite study in five geographic regions of the United States
Source: Front Vet Sci. 2024 Jul 22;11:1435630. doi: 10.3389/fvets.2024.1435630 (PMC11299491; doi:10.3389/fvets.2024.1435630)
Supplement: Supplementary file 1 [file Data_Sheet_1.pdf]

Pet Owner Consent Form

Field assessment of potential exposure of dogs to leptospirosis by measuring titers in dogs
(study identifier)

1. **Purpose of survey:** To serologically screen for potential exposure to leptospirosis in the area.

2. **Purpose of form:** I, the undersigned, consent to participate in a research survey conducted at the _____(prefilled)_____________ for my dog (individually identified below).

3. **Survey requirements:** I understand and agree that my dog will participate in this field survey. I represent and warrant that I am the owner of the dog described below with unencumbered rights to (i) execute this form and (ii) enroll my dog in this survey. As part of the participation, I: (i) agree to allow my dog to have blood drawn, (ii) understand that components of my dog's blood will be used to assess the presence of leptospirosis, (iii) understand that the results of this survey may be submitted to a regulatory agency and published but will not be submitted to me personally, and (iv) agree to allow my dog's serum sample to be sent to a laboratory for testing.

4. **Release:** This research survey has been fully disclosed. I have been given and understand all of the details of the survey, and all of my questions have been addressed to my satisfaction. I hereby agree to release and hold harmless Merial, Inc. and its parent company, the Investigator, and the participating clinic listed above, as well as their respective officers, agents, employees, and contractors from all liability, including but not limited to personal injury, and/or property damage, for any reason. By execution of this form, I understand and agree to all of the provisions of this form, and a copy of this form has been provided to me.

Dog's name: ____

Owner's printed name: ____

Owner's signature: __________________________________ Date: _________________

Clinic Verification (for clinic staff use only)

Clinic name: _____(prefilled)_______________________________________

Clinic street address, city, and state: _________(prefilled)__________________

Investigator's printed name: ____

Investigator's signature: ______________________________ Date: _______________
