# Supplementary material for: Field assessment of potential exposure of dogs to leptospirosis by measuring antibody titers in dogs: a multisite study in five geographic regions of the United States
Source: Front Vet Sci. 2024 Jul 22;11:1435630. doi: 10.3389/fvets.2024.1435630 (PMC11299491; doi:10.3389/fvets.2024.1435630)
Supplement: Supplementary file 2 [file Data_Sheet_2.pdf]

Appendix 2: Individual demographic information and serovars for Leptospira-positive samples by region

Key: ictero = icterohaemorrhagiae
grippo = grippotyphosa

Mid-Atlantic US					
locale & state	Sample number	Dog age (years)	Dog weight (lbs)	Dog sex	Leptospira serovars to which antibodies were detected
SW Raleigh, NC area	MAA-04	6	73.2	M	ictero, grippo, bratislava
	MAA-06	4	69.8	SF	pomona, autumnalis
S Norfolk, VA area	MAB-02	1.58	14	SF	bratislava
	MAB-38	10	72.5	SF	ictero, grippo
	MAB-44	4	55	F	bratislava
S Winston-Salem, NC area	MAC-19	5	57.4	NM	bratislava
Lexington, NC area	MAD-03	3.66	13	NM	bratislava, sejroe
N Norfolk, VA area	MAE-03	12.17	62	NM	bratislava
	MAE-06	7.5	86.9	SF	canicola,bratislava
	MAE-09	7.75	70.8	NM	bratislava
	MAE-13	12.5	8.5	SF	ictero
	MAE-19	5.33	61	NM	grippo
Burlington, NC area	MAF-11	3.58	16	F	bratislava
	MAF-28	4.75	67.2	NM	autumnalis
	MAF-30	6	115	SF	pomona, grippo, bratislava, autumnalis
Jacksonville, NC area	MAG-06	2	41	SF	grippo
	MAG-10	2	40	SF	ictero

Midwest US					
locale & state	Sample number	Dog age (years)	Dog weight (lbs)	Dog sex	Leptospira serovars to which antibodies were detected
SW Madison, WI area	MWB-01	4.66	28.3	NM	pomona, autumnalis
	MWB-18	2.92	60	NM	bratislava
	MWB-22	1.6	62	SF	pomona, ictero, grippo, bratislava, autumnalis
	MWB-24	12.83	14.5	SF	pomona, ictero, grippo, autumnalis
	MWB-39	10.25	56.5	SF	ictero
	MWB-43	5	136	SF	pomona
	MWB-49	1.83	90	NM	grippo
NW Milwaukee, WI area	MWC-05	5	63	NM	ictero
	MWC-07	2	33	SF	pomona, ictero, grippo, autumnalis
	MWC-14	2	10.5	SF	ictero
	MWC-24	9	19	SF	pomona, grippo, bratislava, autumnalis
N Central WI area	MWD-11	2.5	71.3	SF	pomona, bratislava, autumnalis
	MWD-25	4.5	112.6	SF	pomona, ictero, bratislava, autumnalis
	MWD-27	10.5	62.4	SF	pomona, ictero, grippo, bratislava, autumnalis
	MWD-35	10.83	64.3	NM	pomona, ictero, canicola, grippo, autumnalis
	MWD-42	13.17	39.2	M	grippo
W Milwaukee, WI area	MWE-02	8.08	39.7	SF	bratislava
	MWE-09	6.17	17.8	SF	pomona, bratislava
	MWE-11	2.5	37.5	SF	ictero, autumnalis
	MWE-14	5.75	63.8	NM	pomona
	MWE-16	1.83	7.2	NM	bratislava
	MWE-23	8	49.3	SF	grippo
Central WI area	MWF-13	5	33	SF	bratislava, autumnalis
	MWF-14	8	16	NM	pomona, grippo, autumnalis
	MWF-25	11	73	SF	pomona, grippo, bratislava, autumnalis
	MWF-37	14	14	NM	bratislava
	MWF-50	3	86	M	pomona, bratislava, autumnalis
	MWF-53	11	71	SF	ictero
	MWF-62	3	44	SF	bratislava
	MWF-70	10	50	SF	pomona, grippo, bratislava, autumnalis
	MWF-71	10	70	SF	pomona, grippo, bratislava, autumnalis
S Central WI area	MWG-13	12.58	76.4	NM	bratislava
	MWG-17	9.08	95.8	SF	grippo, bratislava
	MWG-23	3	53.5	SF	pomona, grippo, bratislava, autumnalis
	MWG-36	13.08	61	NM	autumnalis
	MWG-39	8.75	58.3	NM	ictero
	MWG-43	5.16	96.3	SF	pomona, ictero, grippo, autumnalis
	MWG-46	6	57.1	NM	autumnalis

Midwest US (cont)					
locale & state	Sample number	Dog age (years)	Dog weight (lbs)	Dog sex	Leptospira serovars to which antibodies were detected
SE WI area	MWH-10	1.5	119	NM	pomona, ictero, grippo, bratislava, autumnalis
	MWH-22	13	30	SF	Grippo
	MWH-37	4	80	F	bratislava, autumnalis
	MWH-38	7	83.5	NM	canicola
	MWH-39	5	60.5	SF	pomona, grippo, autumnalis
	MWH-45	9	80.6	NM	ictero, canicola, grippo
NE Illinois area	MWI-01	8	18	M	canicola
	MWI-03	3	8.5	SF	bratislava
	MWI-05	10	10	SF	pomona, ictero, canicola, grippo, autumnalis
	MWI-15	6	53	NM	bratislava
	MWI-18	6	57	NM	grippo, bratislava
	MWI-22	9	68	SF	ictero
	MWI-42	6	15	NM	ictero
	MWI-45	2	19.2	NM	pomona, canicola, autumnalis
	MWI-46	8	19.8	NM	pomona, grippo, autumnalis
	MWI-47	8	30.3	NM	autumnalis
	MWI-49	5	64	NM	bratislava

Northeast US					
locale & state	Sample number	Dog age (years)	Dog weight (lbs)	Dog sex	Leptospira serovars to which antibodies were detected
SE Massachusetts area	NEA-01	11	44.5	NM	autumnalis
	NEA-05	7	13.7	NM	ictero
	NEA-13	5	84.8	SF	pomona, grippo, autumnalis
	NEA-21	13	45	NM	ictero
	NEA-23	9	16.8	NM	bratislava
	NEA-25	9	70	NM	grippo
	NEA-37	2	55.5	M	bratislava
	NEA-43	4	58	NM	ictero
	NEA-45	5	114	F	bratislava
	NEA-48	1.5	56	M	pomona, ictero, grippo, autumnalis
Central NY State area	NEB-08	3	63.7	SF	autumnalis
	NEB-14	4	50	SF	grippo, bratislava
	NEB-23	2.42	11.69	SF	bratislava
	NEB-27	5.17	65.1	SF	pomona, canicola, grippo, bratislava, autumnalis
	NEB-43	3.5	11.6	NM	bratislava
	NEB-48	9.75	16.25	NM	canicola
NE Rhode Island area	NEC-06	2.75	66.7	SF	pomona, autumnalis
	NEC-11	1.5	91.5	NM	pomona, autumnalis
	NEC-18	4	148	NM	autumnalis
	NEC-19	4.5	68.1	NM	pomona, autumnalis
	NEC-32	5.17	58.6	NM	grippo, bratislava
SE NY State area	NED-09	6	65	SF	pomona, bratislava, autumnalis
	NED-14	13	32	SF	pomona, ictero, grippo, autumnalis
	NED-23	8	47	SF	autumnalis
	NED-24	3	79	NM	pomona, bratislava
	NED-36	11	61	SF	grippo
	NED-39	10	105	SF	ictero
	NED-44	1	16	M	autumnalis
West Boston, MA area	NEF-04	5.42	42.6	NM	ictero, canicola, grippo
	NEF-12	8.83	21.5	NM	pomona, bratislava, autumnalis
	NEF-17	4.5	55.8	NM	pomona
	NEF-18	6.58	101	NM	ictero
	NEF-22	4.58	46.6	SF	bratislava
East-Central MA area	NEG-11	11.17	90	M	bratislava
	NEG-18	11	45.5	F	autumnalis
	NEG-19	6.17	35.9	M	ictero
	NEG-26	7	80	SF	bratislava
North Albany, NY area	NEH-04	15	21.4	NM	pomona, ictero, autumnalis

South-Central US					
locale & state	Sample number	Dog age (years)	Dog weight (lbs)	Dog sex	Leptospira serovars to which antibodies were detected
SE Houston, TX area	SA-05	6	14.1	F	canicola
	SA-18	10	11	NM	pomona, ictero, grippo
	SA-19	9	34.4	NM	bratislava
	SA-32	5	62	MN	grippo, bratislava
	SA-33	3	12.3	NM	grippo, bratislava
	SA-38	2	60.9	NM	grippo
West Houston, TX area	SB-30	9.83	23.8	SF	grippo
	SB-34	6	67.8	SF	grippo
	SB-36	12	86.1	SF	grippo
NW Houston, TX area	SC-15	2	47	SF	autumnalis
	SC-34	7	93	SF	pomona, grippo, bratislava
	SC-36	2	108.6	NM	grippo
	SC-38	13	22.2	SF	grippo, bratislava
	SC-50	5	17	SF	grippo, bratislava
Central Houston TX, area	SD-01	12	39	SF	grippo, bratislava
	SD-07	12	32	NM	canicola
	SD-11	9	54	SF	grippo, autumnalis
	SD-32	3	80.8	NM	autumnalis
	SD-33	10	55	SF	autumnalis
	SD-37	13	17.8	SF	grippo, autumnalis
	SD-55	5	22	NM	bratislava
College Station, TX area	SE-01	3	57.3	NM	canicola
	SE-04	2	9	SF	pomona, ictero, bratislava, autumnalis
	SE-06	2	47	NM	pomona, ictero, bratislava, autumnalis
	SE-11	6.5	46.5	SF	bratislava
	SE-48	4	67	SF	sejroe
SE Houston, TX area (2)	SF-04	4	15.1	NM	bratislava
	SF-14	3	83.4	NM	grippo
North Houston, TX area	SG-08	2	41	SF	autumnalis
	SG-19	6	61.4	NM	bratislava
	SG-31	13	7	SF	canicola, autumnalis
	SG-45	11	50.4	M	grippo, bratislava
SW Houston, TX area	SH-20	5	19.1	M	bratislava

Southwest US					
Clinic name, city, & state	Sample number	Dog age (years)	Dog weight (lbs)	Dog sex	Leptospira serovars to which antibodies were detected
S San Diego, CA area	WD-33	10	23	M	bratislava
W San Diego, CA area	WE-01	7	21.5	M	ictero, grippo
	WE-02	2	55.1	NM	bratislava
	WE-17	4	39.6	NM	ictero
Ventura, CA area	WG-12	12	14	F	bratislava
	WG-13	7	7.7	F	bratislava
	WG-40	6	19.8	M	ictero
NW San Diego, CA area	WH-13	13	50	M	grippo
	WH-23	2	46.5	SF	grippo
	WH-39	13	40	SF	ictero
	WH-48	5	17.9	SF	pomona, ictero, grippo, autumnalis
